# Recognizing Hong Kong Chiropractors’ Sick Leave Authority: Valuing a Conservative Approach to Workers’ Compensation

**DOI:** 10.7759/cureus.36879

**Published:** 2023-03-29

**Authors:** Andy Fu Chieh Lin, Eric Chun-Pu Chu, Valerie K Chu, Vincent Chan, Albert C Leung, Rick P Lau, Kary K Lam, Jacky C Yeung, Kingsley Leung, Lucina Ng

**Affiliations:** 1 Chiropractic and Physiotherapy, New York Medical Group, Hong Kong, HKG; 2 Executive Office, Chiropractic Doctors Association of Hong Kong, Hong Kong, HKG; 3 Chiropractic, Private Clinic, Hong Kong, HKG

**Keywords:** health public, public health care, hong kong chiropractor, chiropractor, public health problems, sick leave, chiropractic therapy, chiropractic management, public health clinics

## Abstract

Although registered under Hong Kong's legislative framework, chiropractors are not able to certify sick leave, restricting the effectiveness of their services for patients with musculoskeletal issues requiring time away from work. This paper explores the evolution of chiropractic regulation in Hong Kong, the growth of the profession, and the tardy recognition of chiropractors' power to issue sick leave certificates. The chiropractic profession and its patients have long lobbied for this authority, but the government has been slow to respond. This document presents a comprehensive analysis of the benefits and restrictions of allowing chiropractors prescriptive authority for sick leave and requests that this change in policy be considered. Developing responsible criteria for chiropractors to prescribe sick leave within their scope of practice could legitimize chiropractic's position in the population's health and interdisciplinary pain care while lowering the burden on injured workers.

## Introduction and background

Not all statutorily registered healthcare professionals in Hong Kong can certify sick leave. According to Hong Kong’s labor laws, under the Medical Registration Ordinance (Cap. 161), only medical doctors or registered Chinese medicine practitioners have this authority [1, 2]. Despite being registered under Hong Kong's legal framework, other medical professionals, such as chiropractors, cannot officially specify the amount of time needed to recover from a health condition. This restricts the ability of patients who rely on such healthcare professionals to obtain necessary sick leave. In order for this to happen, the government would need to amend the current legislation to recognize sick leave certificates provided by other healthcare professionals. So far, these changes have been slow to arise for chiropractors, despite the advocacy of both the profession and patients.

The history of chiropractic in Hong Kong precedes the outbreak of World War II [3]. The enactment of the Chiropractors Registration Ordinance [3] in 1993 marked the beginning of statutory oversight of chiropractors. This Ordinance established the Chiropractors Council of Hong Kong and made Hong Kong the first Asian country to recognize chiropractic as a regulated health profession [3]. Legally, only chiropractors registered with the Council are permitted to practice chiropractic and use the term "registered chiropractor." [3] Chiropractors in Hong Kong are legally authorized to diagnose and treat musculoskeletal conditions. As conservative spine specialists and primary care practitioners, numerous studies have reported that chiropractors in Hong Kong provide non-invasive care for prevalent neuromusculoskeletal conditions and injuries [4-23]. However, their inability to officially certify patients' incapacity limits the utility of chiropractic services. Granting chiropractors prescriptive authority to authorize necessary sick leave could more comprehensively support their management of common musculoskeletal diagnoses and facilitate a safe return to function for patients.

Chiropractors in Hong Kong have long advocated for the authority to certify sick leave. Since being formally recognized in 1993, the profession has grown, but chiropractors' sick leave certificates remain unrecognized. By contrast, registered Chinese medicine practitioners have been able to certify sick leave since 2006 [24]. The Hong Kong government has been slow to take action on the authorization of chiropractors' sick leave certificates. An interdepartmental government working group was formed in 2005 to study this issue but has not reached a conclusion after nine meetings over four years [24]. The working group is evaluating factors such as chiropractic's scope of practice, training, codes of practice, and international practices [24]. Without a clear timeline or commitment to making a definitive decision, patients requiring time away from work for chiropractic treatment of musculoskeletal conditions may face difficulties obtaining the necessary sick leave [24]. Currently, injured workers seeking chiropractic care are obliged to make another trip to see a medical doctor for a sick leave certificate. This arrangement unnecessarily increases the burden on the patient in terms of money, time, stress, and effort.

The criteria for recognizing sick leave certificates likely considers the scope of practice and training of the prescribing profession. Responsibility for these criteria is likely to fall under the relevant government department responsible for overseeing each profession. Given that the Employees' Compensation Ordinance was made for a complete employee compensation system, it approves chiropractic treatment for work injuries [24]. However, the rationale for not recognizing chiropractors' sick leave certificates is unclear [24]. Our study provides a broad overview of the benefits and limitations of recognizing chiropractors' sick leave authority.

## Review

Chiropractic service has been widely used in Hong Kong

Chiropractors in Hong Kong are trained to diagnose and treat musculoskeletal and neurological conditions amenable to chiropractic care [3]. Chiropractic is a distinct system of healthcare that focuses on the structure and function of the body, particularly the spine and nervous system. Chiropractors use manual adjustments and other such techniques to treat dysfunctional spinal joints and supporting soft tissues to enhance function and reduce pain [3]. According to the results of a 2021 survey, 90.41% of chiropractors in Hong Kong promote spine and joint health, and 87.67% treat neuromusculoskeletal disorders [25]. The majority of chiropractic patients seek treatment for musculoskeletal conditions [26]. The proliferation of 185 chiropractic clinics in 27 mass-transit railway (MTR) stations [27, 28] in Hong Kong indicates the strong public demand for this conservative approach to managing musculoskeletal conditions.

While greater awareness and understanding of chiropractic would facilitate broader acceptance, policy changes could also further legitimize the profession. People are educated about the efficacy of chiropractic care through a variety of channels, including social media, advertisements, informational seminars, and online resources [29-31]. Chiropractors have provided spinal education in 348 local schools, training over 1,000 teachers, and reaching over 400,000 students [29, 32]. As a result, chiropractic organizations in Hong Kong received 31 World Spine Day Awards, the largest global public health event dedicated to promoting spinal health and well-being, between 2014 and 2022 [29-31]. Moreover, the United Nations Sustainable Development Goals (SDG) for Hong Kong has recognized Hong Kong’s chiropractic organization, New York Medical Group, with regard to SDG 3, which seeks to promote spinal health and well-being for all [33]. The United Nations Children's Fund Hong Kong (UNICEF) has also recognized Eric CP Chu, a chiropractor who has influenced corporations in promoting breastfeeding [34]. These awards demonstrate the significant increase in chiropractic awareness in Hong Kong. Recognizing chiropractors' authority to certify sick leave under appropriate conditions would serve to further affirm the value of chiropractic care.

The evidence of chiropractic's acceptance in Hong Kong lies in its inclusion in major insurance plans and government health programs. Most private insurance companies cover chiropractic services, with injured employees permitted to claim chiropractic treatment under the Employees' Compensation Ordinance. In fact, chiropractic services accounted for 2.3% of total claims in this area in 2019 [35]. The fact that both insurers and the government recognize and pay for chiropractic care reflects the legitimacy of this approach and its popularity among Hong Kong’s population as a conservative option for musculoskeletal conditions. Chiropractic is also included in the Mainland and Hong Kong Closer Economic Partnership Arrangement [36]. The Elderly Health Care Voucher Scheme subsidizes the use of designated private healthcare providers for older patients, including chiropractors [37]. The inclusion of chiropractic under various health systems and plans provides further support for the motion to grant chiropractors authority to certify sick leave.

Chiropractic as an effective and low-risk therapy

Chiropractors in Hong Kong utilize manual techniques such as spinal manipulation and soft tissue mobilization to address musculoskeletal dysfunction and reduce pain [4-23]. According to a 2013 survey undertaken by the Census and Statistics Department of the Hong Kong Special Administrative Region, more than half (53.6%) of the 33,700 individuals who received chiropractic care had previously tried other forms of treatment. These included medical specialists (22%), general practitioners (24%), physiotherapists (18%), and Chinese medicine practitioners (18%) [38], with less than 10% of respondents indicating that chiropractic treatment was completely ineffective [38]. In addition, a large adverse events study in Hong Kong that analyzed unique patients and covered 960,140 treatment sessions revealed that the incidence of severe adverse events arising from chiropractic treatment was only 0.21 per 100,000 treatments, indicating that these complications were uncommon and reassuringly minimal [39].

Chiropractic care offers a non-drug-oriented approach to pain relief that could reduce the reliance on prescription analgesics, including opioids [40]. Considering the risks of addiction, overdose, and other harmful effects of opioid pain medications, conservative manual treatment is an important treatment option for musculoskeletal pain [40]. Chiropractic manipulation and soft tissue techniques are non-invasive and produce minimal side effects, and thus may help to lower the significant risks associated with pharmacotherapy [39]. By decreasing the use of opioids and other medications, expanded access to chiropractic services could help curb the growth of the opioid prescription epidemic. Integrating chiropractors alongside medical doctors in pain management could provide a more comprehensive set of options for improving patient outcomes and overall safety.

How issuing sick leave certificates would enhance chiropractic's role

The ability of chiropractors to certify sick leave would affirm chiropractic as an essential medical treatment and establish chiropractors as the primary spine and joint care providers. Chiropractors cannot currently officially authorize time needed away from work for musculoskeletal recovery, which limits the recognition of the value of chiropractic care. Were they granted the prescriptive power to indicate incapacity and authorize necessary sick leave, chiropractors would have a formal means of validating the importance of the treatment they provide. This authority could elevate the status of chiropractic as a profession, supporting its growth and integration into the healthcare system. Wider acceptance of chiropractic's role in managing back and joint conditions could increase patients’ access to the important conservative techniques this discipline offers.

The ability to certify sick leave would allow chiropractors to more comprehensively oversee patient treatment and recovery [24]. By indicating the necessary time away from work or activities that can exacerbate a musculoskeletal condition, chiropractors could construct customized care plans tailored to patients' specific pain or impairment and job or functional demands. The power to formally specify a gradual return to full activity as conditions improve would enable chiropractors to safely control the pacing of recovery and minimize the risk of symptom recurrence or exacerbation. These measures would allow chiropractors to appropriately manage patients’ recovery and endorse a stepwise return to full function, and they would further support the utilization of their services to address prevalent spinal and joint issues.

Thus, the ability to certify sick leave would elevate the status of chiropractic care and potentially increase its use. With this legally recognized authority, chiropractors would have a formal means of demonstrating the medical value of their services. This could build public confidence in chiropractic and dispel misconceptions regarding its legitimacy and effectiveness. For the chiropractic profession, the ability to prescribe sick leave would underscore the role of chiropractors as spine and joint specialists and would help to attract high-quality practitioners to the field. If more people seek chiropractic treatment for musculoskeletal conditions, this may reduce their reliance on medications, advanced imaging, or surgery. Granting chiropractors sick leave authorization could thus benefit both public health as a whole and the chiropractic profession itself.

Potential concerns and recommendations to address

While an interdepartmental working group was appointed to evaluate recognizing chiropractors' sick leave authority, it has not yet recommended this change [41]. The working group considered chiropractic practice in Hong Kong, evidence of treatment efficacy, practices in other countries, and stakeholder views [41]. So far, this evaluation process has lasted years without reaching a resolution. The government should determine whether chiropractors' sick leave certificates can be recognized responsibly based on the conditions they treat and establish guidelines and safeguards accordingly. Patients would benefit from chiropractors being granted the ability to officially certify necessary time away from occupational tasks relating to common musculoskeletal conditions. Rather than deny this authority merely because of concerns, a prudent trial could assess its feasibility if the government committed to responsible application. Henceforth, we will discuss the potential concerns and recommendations to be addressed.

Chiropractors in Hong Kong have long fought for the right to certify sick leave, arguing that this authority would legitimize their role as spine and joint specialists. However, the government is cautiously evaluating this proposal due to concerns about the potential overuse or misapplication of sick leave certification given chiropractors' focused scope of practice [41]. A working group continues to study the issue, considering factors such as chiropractic's regulatory framework and training, community acceptance, and implications for stakeholders [41]. Not all medical professions can currently certify sick leave, and practices vary internationally. Thus, this working group is charged with determining if and how to grant chiropractors this authority in order to benefit Hong Kong's healthcare system and improve workplace injury management.

Lack of International Practices

While practices regarding chiropractors' roles in labor systems vary internationally [42], this should not preclude Hong Kong from recognizing chiropractors' authority to grant sick leave certificates if it would benefit patients and the healthcare system. The government is carefully evaluating this issue given the differences in how countries govern chiropractic practices. However, it is clear that with responsible guidelines, chiropractors can appropriately certify necessary time away from work for the common musculoskeletal conditions they treat. The granting of this authority would affirm chiropractic's value in managing spinal and joint issues that directly affect people’s ability to function. Rather than dismissing this change in response to global variances in chiropractors' labor-related roles, Hong Kong should determine whether and how to implement it based on local needs and chiropractic's observed contributions.

Fewer Reported Employees Managed by Chiropractors

While relatively few injured employees have sought chiropractic treatment that is covered under Hong Kong's compensation system [42], this reflects limited public knowledge of chiropractic's value rather than an innate lack of value. Increasing awareness of the safe and effective approaches offered by chiropractic for managing back, neck, and joint pain would allow more patients to benefit from this conservative treatment option for musculoskeletal conditions. Granting chiropractors the authority to administer sick leave certificates would help to raise the profile of chiropractic care and its applications by educating the public and other stakeholders on how chiropractors can contribute to effective pain management and recovery. Rather than denying this authority because of its limited current usage, this recognition could support the growth of underutilized healthcare services.

No Tertiary Institutions Providing Chiropractic Training

While Hong Kong lacked tertiary chiropractic education and neutral certification authorities beyond the chiropractic profession itself in 2005 [42], the McTimoney College of Chiropractic in the United Kingdom now offers a four-year Master of Chiropractic program in Hong Kong. This program is designed to meet the chiropractic care needs of the Hong Kong community and serve the broader Hong Kong population. It is the first chiropractic course to be made available in Hong Kong and the Greater China region and aims to equip chiropractors with the knowledge and skills necessary to provide high-quality chiropractic care [43]. Twenty-nine percent of chiropractors currently practicing in Hong Kong hold continuing education in advanced degrees [25]. The Education Institute can serve as a tertiary institution by providing standards and guidelines that define objective criteria for medically necessary sick leave, thereby minimizing disputes between chiropractors and employers or insurers. Chiropractic physicians can be held accountable in the same manner as other professionals authorized to certify sick leave. Instead of denying this authority outright, owing to structural differences with other medical professions, the government could investigate safeguards that would allow chiropractors to responsibly certify sick leave if this change would benefit patients.

New Legal Obligations

Before recognizing chiropractors' ability to grant sick leave certificates, the government and other stakeholders should understand the conditions that chiropractors treat and the relevant criteria for determining necessary time away from work. This understanding would clarify chiropractors’ responsibilities and obligations, thereby minimizing the likelihood of disputes. Through education on chiropractic's conservative approach to treating musculoskeletal conditions, appropriate sick leave could be prescribed for conditions within the scope of chiropractors' expertise. Rather than denying chiropractors the authority to grant sick leave due to current limitations in stakeholder knowledge [44], the government could instead focus on raising awareness and determining feasible safeguards and guidelines to enable this authority to improve Hong Kong's management of back and joint problems that impact function.

Sick leave guideline for chiropractors

To ensure responsible use of certified sick leave, chiropractors could be authorized to only certify time away from work relating to musculoskeletal conditions within their officially recognized scope of practice [45]. The Chiropractic Doctors Association of Hong Kong (CDAHK) is the largest chiropractic organization [27], which has already provided a Sick Leave Guideline for Chiropractors in 2013, determining when sick leave is medically necessary for common conditions treated by chiropractors; this could be utilized to form objective criteria for chiropractor-authorized sick leave [45]. Chiropractors could be subject to the same oversight and auditing requirements as other professions that possess sick leave authority to confirm the appropriate application of sick leave. Operating within such parameters and with a high level of accountability, chiropractors would be able to certify sick leave for the conditions they are trained to treat while minimizing the risks of overprescribing or misapplying this authority. If chiropractors in Hong Kong are granted the ability to certify sick leave, clear guidelines surrounding professional integrity can alleviate concerns regarding how this responsibility will be handled.

Should chiropractors be granted the authority to certify sick leave, guidelines should ensure the evidence-based application of this responsibility. With parameters grounded in the conservative scope of chiropractic practice and training, chiropractors can effectively support recovery from common musculoskeletal conditions through safe, patient-centered care. By objectively defining when incapacity necessitates time away from occupational tasks, the misapplication or overutilization of sick leave authority can be minimized. With appropriate oversight and professional accountability, chiropractors can make a valuable contribution to Hong Kong’s healthcare system and public health overall through the administration of prudent sick leave certification. This can be an important aspect of focused specialty care for spinal and joint conditions.

## Conclusions

Recognizing chiropractors' authority to certify sick leave would merit considering strategically expanding access to conservative musculoskeletal care. Although concerns regarding the overuse or misapplication of this authority are valid, responsible guidelines can help enable chiropractors to accurately prescribe the necessary time away from work for conditions within their scope of practice. With appropriate professional standards and levels of oversight, this certification power could legitimize chiropractic's role in serving population health and providing multidisciplinary pain management. Rather than denying chiropractors an opportunity to formally support patient recovery through officially authorized sick leave and augmenting the burden of the injured workers, policymakers could trial this change by confining chiropractors' certification ability to specific, evidence-based clinical criteria.
